# Value of training on motivation among health workers in Narok County, Kenya

**DOI:** 10.11604/pamj.2016.23.261.8414

**Published:** 2016-04-29

**Authors:** George Osoro Momanyi, Maureen Atieno Adoyo, Eunice Muthoni Mwangi, Dennis Okari Mokua

**Affiliations:** 1 Department of Health Systems Management, Kenya Methodist University, Nairobi, Kenya; 2 Department of Biological Sciences, Egerton University, Njoro, Kenya

**Keywords:** Human resource interventions, training, continuing professional development, health worker motivation, health systems, Narok County

## Abstract

**Introduction:**

Training, as an additive human resources intervention is decisive to organizational performance. Employees require constant update of formal and informal knowledge alongside positive attitudes that have been defined as necessary in motivation leading to effectiveness in performance hence workplace training is tied to achieving organizational aims and objectives. The objective of this study was to determine the influence of training on motivation among health workers in Narok County, Kenya.

**Methods:**

A cross-sectional study utilizing a self administered questionnaire, targeting 237 health workers and 21 health managers was used. Data analysis was done using SPSS version 21 using descriptive statistics. Factor analysis was done on the training perception in relation to motivation.

**Results:**

Majority of the respondents rated their motivation between 7 and 9 in the current health facility (35.4%), Sub-county (33.8%) and County (32.9%) with the median motivation level of 5. Majority of health workers 194 (81.9%) had received a form of training, of whom 191 (98.5%) indicated that on-job training was relevant to their tasks and that it motivated 192 (99.0%) of them to perform better due to coining skills to motivation. Training significantly predicted general motivation (p-value = 0.013), job satisfaction (p-value = .001), intrinsic job satisfaction (p-value = .001) and organisational commitment (p-value <.001).

**Conclusion:**

The researchers concluded that there is a relationship between training and motivated health workforce in Narok County and recommended strengthening of current training initiatives by ensuring trainings are more regular and involvement of health workers in discussing their career development prospects.

## Introduction

Training in a work organization is essentially a learning process, in which learning opportunities are purposefully structured by the management and training staff working in collaboration [1]. The aim of the process is to develop in the organization's employees the knowledge, skills and attitudes that have been defined as necessary to motivate them to effectively perform their work and hence the achievement of organizational aims and objectives. Employers therefore depend on the quality of their employees′ performance to achieve organizational aims and objectives. Training has been used to upgrade skills and knowledge in the health care sector in resource poor settings as off-site training courses and seminars. As an intervention to motivate and improve practices of health providers, it has not proven to be very effective due to a lack of problem analysis and training-needs assessment [2, 3]. There is a notion that training has an association with disparities reflected in training content and skills needed in the work environment, as well as methods involved in its delivery [4]. Other than the methods of delivering training, actual access of training among health workers vary [5]. In Kenya it has been observed that health workers in the public sector have limited access to training and further qualifications is limited or granted in line with reasons that are not equitably available or merit-based, this can likewise have detrimental consequences for the motivation effect of training as a tool of HRM [4]. Inadequate knowledge, skills and inappropriate attitudes can all form obstacles to good health care. As a result, advances in insights into treatment and diagnosis, as well as changes in roles and responsibilities, require continuous professional development among health workers and this serves as a motivator. According to the World Health Organization [6], a lifelong learning process must be developed at the start of a professional career in the health sector in order to realize better health benefits. The benefits of training are many: improves morale of employees, helps the employee to get job security and job satisfaction, helps refreshing past knowledge and practice, and helps to identify and correct mistakes [7, 8].

This study was necessitated by the relatively sub optimal performance on key health indicators related to maternal and child health [9]. Health workers are important human resources (HR) that will contribute to realizing the health part of the Millennium Development Goals (MDGs) [10]. In Kenya, the health sector is pivotal to achievement of vision 2030 [11] and as a third world country with limited resources for providing quality health services for all of its citizens, success in providing quality health care should involve developing innovative strategies to meet the health objective. Part of these innovations is to develop strategic human resource management (HRM) interventions proven to motivate health workers in order to ensure prudent use of the minimal resources available. At the time of the study, the authors were unaware of any published reports on the influence of training on motivation among health workers in Narok County, Kenya. Considering that training is an important input in HRM which has an impact on quality healthcare delivery and contributes to strengthening the HR pillar of every health system [12], the study was carried out to document the status of the influence of training amongst health workers serving in public health facilities in Narok County, Kenya. In the current devolved health care system in the country which is relatively a new dispensation in healthcare, these findings would contribute in identifying if training is really a priority area of intervention in motivating health workers thereby providing evidence to County health managers on appropriate investments known to improve performance in order to maximize on resources available for greater health benefit.

## Methods

**Study area:** The study was conducted in health facilities in Narok North and Transmara West sub counties, in Narok County. There had been no documentation on the effectiveness of training interventions in Narok County.

**Study design and procedure:** A cross-sectional survey was used to document the status of the influence of training as a HRM intervention on motivation of the health workers in Narok County, which targeted 237 respondents among the health workers of all professional cadres and 21 respondents from the health managers. Measurable variable was training (availability, training perceptions and influence of training on performance) which formed the independent variable while the dependent variable was motivated health worker. To assess the motivation levels among the health workers, the researcher adopted two approaches, one was a 10-point self-rated scale which was used to measure motivational levels for working at the current institution, Sub-County and County while the other was a multiple question on constructs relating to motivation i.e. general motivation, job satisfaction, intrinsic job satisfaction, organizational commitment, conscientiousness, timeliness and attendance. To assess the relationship between the training intervention and health workers’ motivation, factor analysis was carried out on the perceptions of training such that the factor with the highest factor loading with Eigen vectors more than one was taken into account. After recoding negatively worded questions, factor analysis was also carried out on the dimensions of motivation such that for each dimension the factor accounting for the highest factor loading with Eigen vectors more than one was taken into account. The Eigen vectors developed for both training and dimensions of motivation were then used for further analysis. In order to test the null hypothesis of no relationship between training and health workforce motivation in Narok County, multivariate regression was carried out involving the developed Eigen values

**Data collection:** Self administered structured questionnaires were used to collect data. The health workers’ questionnaire was organized to capture data on social demographic characteristics, motivation level of health workers and the training intervention areas (availability, training perceptions and influence of training on performance). The health managers’ questionnaire majorly contained two sections: social demographics and indicators for improved performance among the health workforce.

**Data management and analysis:** At the end of each interview the filled questionnaire was cross checked for completeness and any missing entries corrected. Data collected was coded, processed and cleaned off current inconsistencies and outliers. Data was analyzed by the use of SPSS (Statistical Package for the Social Sciences) version 21 as per the specific research questions and subjected to descriptive analysis using frequencies and percentages.

**Ethical consideration:** Permission to conduct research was sought from the County Director of Health. The researcher provided full information about what the research entailed and ensured participants were competent to give consent. The questionnaires were administered with duly obtaining consent of the participant. Participants’ privacy was maintained by ensuring that they were not exposed to public when filling questionnaires. Anonymity of respondents was assured by concealing their identity and research data was kept confidential for research purposes only. The study was conducted by full adherence of the Scientific and Ethics Review Committee of Kenya Methodist University.

## Results

**Figure 1:**
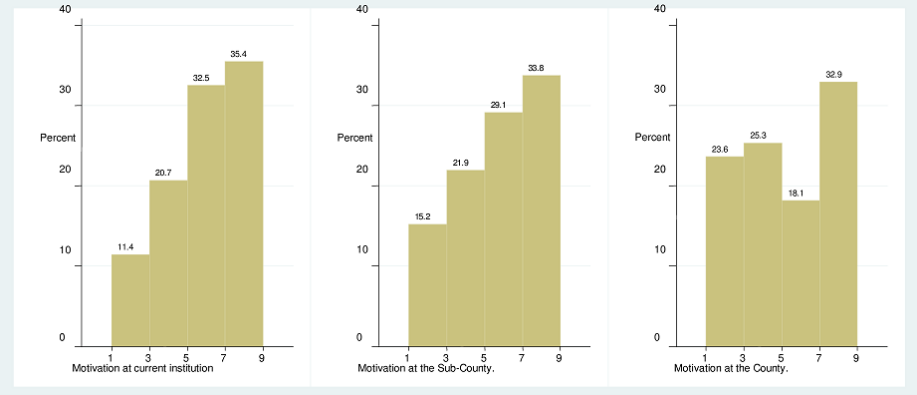
Motivation level at current facility, Sub-County and County

**Motivation level of health workers:** On a scale of 1 (fully demotivated) to 10 (fully motivated), majority of the respondents rated their motivation as between 7 and 9 in the current health facility (35.4%), Sub-county (33.8%) and County (32.9%) as shown in Figure 1. The median motivation level was rated as 5 (implying averagely motivated) at the current health facility, Sub-county and County. There was significant difference in the motivation levels at the current health facility, sub-county and County (Friedman test p-value < .001). Based on a scale of 1 (strongly agree) to 5 (strongly disagree), the respondents centrally scored as follows on multi-item motivation constructs: on general motivation respondents disagreed that they only do their job so as to get paid at the end of the month. On overall the respondents agreed that they were very satisfied with their jobs. On organisational commitment, respondents disagreed that they felt very little commitment to the health facility they were based in. On conscientiousness, respondents strongly disagreed that they could not be relied on by their colleagues at work. On timeliness and attendance, respondents agreed that they were always punctual at coming to work, see Table 1.

**Table 1:** Motivation constructs

Construct	Questions	Median
General motivation	These days, I feel motivated to work as hard as I can	2
	I only do this job so that I get paid at the end of the month	4
	I do this job as it provides long term security for me	3
Job satisfaction	Overall, I am very satisfied with my job	2
	I am not satisfied with my colleagues in my ward/health facility	4
	I am satisfied with my supervisor	2
Intrinsic job satisfaction	I am satisfied with the opportunity to use my abilities in my job	2
	I am satisfied that I accomplish something worthwhile in this job	2
	I do not think that my work in the hospital is valuable these days	4
Organizational commitment	I am proud to be working for this hospital	2
	I find that my values and this hospital's values are very similar	2
	I am glad that I work for this facility rather than other facilities in the country	2
	I feel very little commitment to this hospital	4
	This hospital really inspires me to do my very best on the job	2
Conscientiousness	I cannot be relied on by my colleagues at work	5
	I always complete my tasks efficiently and correctly	2
	I am a hard worker	1
	I do things that need doing without being asked or told	1
Timeliness and attendance	I am punctual about coming to work	2
	I am often absent from work	5
	It is not a problem if I sometimes come late to work	5

**Figure 2:**
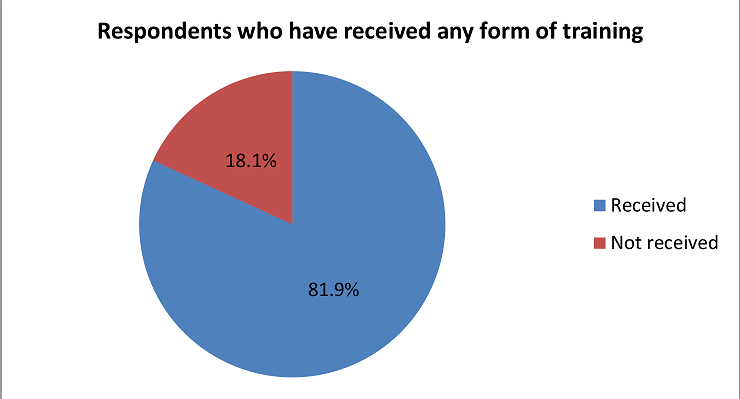
Availability of training according to study participants

**Influence of training to motivation Availability of training:** As shown in Figure 2 majority 194 (81.9%) of the respondents had received a form of training within the health facilities they were based in.

**Training perceptions:** Majority of the respondents thought that the on-job training they had received was relevant 191 (98.5%) to their day-to-day duties in the health facilities and that it motivated 192 (99.0%) them to perform better at their work places, see Table 2. Majority of the respondents agreed that the organisation had staff training and development policy 127 (53.6%), opportunities for career development existed in the organisation 130 (54.9%), appropriate training was conducted to ensure health workers carry out their duties well 154 (65%), in-service training provided was adequate in dealing with existing skills gap 107 (45.1%), less competent health workers were provided with necessary support to improve their knowledge and skills 116 (48.9%) and that health care workers participate in identifying their career development needs 111 (46.8%), refer to Table 3. However, majority disagree that job refresher courses were provided on regular basis 119 (50.2%) and in the last 6 months their supervisors discussed their career development prospects with them 153 (64.6%), see Table 3.

**Table 2:** Training relevance and its influence on motivation

Training dimensions	Perceptions	Frequency (n = 194)	Percent
Relevance	Relevant	191	98.5
	Irrelevant	3	1.5
Motivation	Motivate	192	99.0
	Did not motivate	2	1.0

**Table 3:** Training perceptions

Training perceptions (% n = 237)	Disagree	Undecided	Agree
This organization has a staff training and development policy	79 (33.3)	31 (13.1)	127 (53.6)
Opportunities exist for career advancement in this organization	79 (33.3)	28(11.8)	130 (54.9)
Appropriate training is conducted to ensure that health care workers carry out their duties well	50 (21.1)	33 (13.9)	154 (65.0)
Job specific refresher courses are provided on a regular basis	119 (50.2)	33 (13.9)	85 (35.9)
The in-service training provided is adequate to deal with the existing skills gap	83 (35)	47 (19.8)	107 (45.1)
Health care workers who are less competent are provided with the necessary support to improve their knowledge and skills	90 (38.0)	31 (13.1)	116 (48.9)
Health care workers participate in identifying their career development needs	83(35.0)	43 (18.1)	111 (46.8)
In the last 6 months my supervisors discussed my career development prospects with me	153 (64.6)	30 (12.7)	54 (22.8)

**Influence of training on performance:** Majority of the respondents agree that the work-related training they received made them make choices consistent with goals assigned to them 109 (56.2%), perform tasks assigned in good speed 93 (47.9%), perform duties assigned accurately 95 (49.0%) and help them go to the greatest extent to achieve goals assigned to them 98 (50.5%), see Table 4. Majority of the health service managers agreed that the work-related training provided made health workers make choices consistent with goals assigned to them 13 (61.9%), perform tasks assigned in good speed 13 (61.9%), perform duties assigned accurately 10 (47.6%) and help them go to the greatest extent to achieve goals assigned to them 11 (52.4%), see Table 5.

**Table 4:** Influence of training on performance (perceptions of health workers)

Training effect (% n = 194)	Strongly agree	Agree	Neutral	Disagree	Strongly disagree
Made me to make choices consistent with the goal assigned	63 (32.5)	109 (56.2)	13 (6.7)	7 (3.6)	2 (1.0)
Made me to perform the tasks assigned in good speed	76 (39.2)	93 (47.9)	19 (9.8)	5 (2.6)	1 (0.5)
Made me to be accurate in performing duties assigned	70 (36.1)	95 (49)	24 (12.4)	4 (2.1)	1 (0.5)
Made me always go to the greatest extent to achieve goals assigned	63 (32.5)	98 (50.5)	22 (11.3)	8 (4.1)	3 (1.5)

**Table 5:** Influence of training on performance (perceptions of Health Service Managers)

Statement (% n = 21)	Strongly agree	Agree	Neutral	Disagree	Strongly disagree
The trainings provided have made health workers to make choices consistent with the goals assigned to them	5 (23.8)	13 (61.9)	2 (9.5)	1 (4.8)	0 (0.0)
The trainings provided have made health workers to perform the tasks assigned to them in good speed	5 (23.8)	13 (61.9)	3 (14.3)	0 (0.0)	0 (0.0)
The trainings provided have made the health workers to be accurate in performing duties assigned to them	4 (19.0)	10 (47.6)	6 (28.6)	1 (4.8)	0 (0.0)
The trainings provided have made health workers to always go to the greatest extent to achieve goals assigned to them	3 (14.3)	11 (52.4)	4 (19.0)	3 (14.3)	0 (0.0)

**Relationship between training and motivation:** Training significantly predicted general motivation (p-value = 0.013), job satisfaction (p-value = .001), intrinsic job satisfaction (p-value = .001) and organizational commitment (p-value <.001); refer to Table 6.

**Table 6:** Multivariate relationship model

Source	Dependent Variable	Type III Sum of Squares	df	Mean Square	F	Sig.
Model	General motivation	22.691^a^	3	7.564	8.297	0.000
	Job satisfaction	39.510^b^	3	13.17	15.684	0.000
	Intrinsic job satisfaction	26.691^c^	3	8.897	9.947	0.000
	Organisational commitment	62.526^d^	3	20.842	28.114	0.000
	Conscientiousness	5.125^e^	3	1.708	1.731	0.161
	Timeliness and attendance	6.497^f^	3	2.166	2.208	0.088
Training	General motivation	5.754	1	5.754	6.313	0.013
	Job satisfaction	10.176	1	10.176	12.118	0.001
	Intrinsic job satisfaction	9.595	1	9.595	10.727	0.001
	Organisational commitment	17.085	1	17.085	23.046	0.000
	Conscientiousness	0.005	1	0.005	0.005	0.944
	Timeliness and attendance	0.034	1	0.034	0.035	0.852

a R^2^ = .096

b R^2^= .167

c R^2^ = .113

d R^2^ = .265

e R^2^ = .022

f R^2^ = .028

*This table depicts the significant predictors of motivation in regard to HRM intervention (training)

## Discussion

This study was undertaken to determine the influence of training on motivated health workforce in Narok County. Overall, there was an average motivation level among health workers in Narok County at their current institution, sub-county and county. These motivation levels were lower as compared to motivation of health workers in Ghana where researchers reported that health workers achieved an overall motivation mean score of 3.65 (out of 5) translating to 7.3 out of 10 [13]. Findings from a study in Zambia reported as high as 60-74% motivation level of health workers in three study districts [14]. Similar low levels of health worker motivation was reported in public health facilities in Thika District, where the results indicated that majority of health workers were demotivated with respect to satisfaction of needs factors and job enrichment factors [15]. Similar findings have also been made in both Kenya and Benin among health workers where it was found that approximately 55% of the respondents did not rate their willingness to do a good job, according to organizational objectives as “rather good”, “high” or “very high” [4]. While measuring motivation using multiple questions on constructs relating to motivation, findings were similar to those made in District hospitals in Kenya where studies reported that the majority of respondents strongly agreed to be hard workers and disagreed that they were often absent from work with many participants describing themselves as demotivated [16]. Similar findings were also made in three districts in Zambia where it was found out that in overall the health workers agreed that they were satisfied with their jobs and were committed to their organization while conscientiousness and timeliness and attendance had the highest scores [14].

These results show that there are wide variations in the general motivation of the health workers across health systems settings. The variations may be explained by differences in levels of factors contributing to motivation as well as differences in health care environments. Different health systems in different locations have different health worker provisions contributing to differences in health worker motivation results. Training which is considered general in evaluating health workers motivation [17] is one of the factors [18] used in their study in Iran. In addition to differences in levels of adoption of HRM interventions in various settings, Kenya is currently going through health care system devolution [19] suggesting that these factors may have different adoption levels hence any motivation level is expected. Results from this study may suggest that the current health care system in Narok County score averagely in the general health care motivation provisions. The respondents were asked if they had ever received any form of training in the current institution, the data collected showed that majority 194 (81.9%) of the respondents had received a form of training within the health facilities they were based in. These findings are consistent with similar study in Malawi that showed access to training to be 94.7% [20]. In Meru County Kenya, it was established that capacity building was usually undertaken through on-job trainings with 85.1% health workers reported to have had on-job training on filling of data collection tools while 10% had received formal classroom training on the same [21]. There is however evidence on the contrary indicating that access to training opportunities among health workers may vary [5]. Therefore, results in this study suggest that current advances in healthcare including insights into treatment and diagnosis as well as changes in roles and responsibilities require continuous professional development among health workers and this serves as a motivator. The current health care system in Narok County has made provisions for on-going training of its health workers. When asked if the on-job training they received was relevant and if it motivated them to perform better, majority of the respondents thought that the on-job training they had received was relevant 191 (98.5%) to their day-to-day duties in the health facilities and that it motivated 192 (99.0%) of them to perform better at their work places. Contrary findings were made in Britain where it was reported that there was more conflict between the idealized perspectives of work gained during training and actual work practice and were less satisfied with their professional organization [22]. Similar findings were made in Kenya where it was found out that among health workers, majority of them prefer to be incentivized by provision of in-service training through continuous medical education [23]. However, a similar study in Kenya and Benin described the effect of training as short-lived or even frustrating [4]. According to these findings, training may constitute a direct means to mobilize the motivational potentials inherent in the professional ethos of health workers. When asked on their training perceptions, results showed similar findings to those made in Uganda where it was found out that most of the health care workers indicated that they had received the training required to succeed in their positions and also agreed that appropriate training was conducted to ensure that they carried out their duties well with some health care workers agreeing that they have participated in identifying their career development needs [24]. In Kenya it was observed that health workers in the public sector have limited access to training and further qualifications is limited or granted in line with reasons that are not equitably available or merit-based, this can likewise have detrimental consequences for the motivation effect of training as a tool of HRM [4]. These findings may suggest that the current health care system in Narok County has put in place an infrastructure to support training of health workers which requires further strengthening. However, there is need for the County health management to ensure sustained effort to make on-going trainings more regular and enhance involvement of health workers in career development prospects in order to sustain gains made.

When comparing perceptions of health workers and health managers’ influence of training on performance, results showed that there was an agreement between health workers and health services managers as to the influence of training on health worker's motivation leading to improved performance. Similar effects were observed in Siaya County where it was found out that there was a relationship between training and employee performance among health workers [7]. In Senegal and Bolivia, training provided improved health workers theoretical competences in respiratory diseases management, but practical competences were not assessed [8]. According to [8] continuous training is important since it helps refreshing past knowledge and practice, helping to identify and correct mistakes. The results suggest that the training intervention undertaken by the Narok County health management has a role in motivating the health workforce thus leading to improved health system performance. While assessing the relationship between training and motivated health workforce, results were significant where general motivation (p-value = 0.013), job satisfaction (p-value = .001), intrinsic job satisfaction (p-value = .001) and organizational commitment (p-value <.001) were all less than the determined significance p ≤ .05. This implies that the null hypothesis that there was no relationship between training intervention and motivated health workforce in Narok County was rejected. The alternative hypothesis that there is a relationship between HRM interventions training and motivated health workforce in Narok County was therefore not rejected.

These results were similar to findings made in public hospitals in New Zealand and United States where perceived access to training, training frequency, motivation to learn from training, benefits of training, and supervisory support for training were positively related to the affective and normative components of commitment [25]. Findings agree with a study conducted in Malaysia which indicated that accessibility and support to training, coupled with the incentive to gain knowledge, training setting and apparent benefits of training were all interrelated with the affective commitment, normative commitment and overall organizational commitment. Similarly, the location of training and the alleged training benefits showed an association with continuance commitment, however there was no association between training availability, support to training and motivation to learn with continuance commitment [26]. Achieving a competitive advantage within the health industry requires strategic HRM that will help align health workers’ activities to the direction, purpose and objectives of the health care system [27]. This study outlined one broad HRM intervention i.e. training and its findings suggested a positive link between training and motivated health workforce from a different angle. The findings also suggested variations depending on different health system environments and level of implementation of training intervention. Training is one of the interventions recommended by the World Health Organization as critical to enhancing health worker motivation [14] and therefore Narok county health system should continuously assess its level of implementation to enhance and exploit the benefits of this relationship.

This study however had some potential limitations which may have affected the results. The study was carried out in two sub-counties in the county. It cannot be assumed that health workers in the other sub counties share similar views. Therefore, the results may not be generalized to the entire county and this is considered a limitation of the present study. The self-administered structured questionnaire used in data collection present a likelihood of respondents rating high on their responses which is an activity that is hard to control and this could have an influence on the results. The studied population comprised of health workers in public health institutions and this may have missed out an interesting analytical angle had the study included health workers from private institutions.

Based on the results of the study, training is already in place and has been shown to enhance health worker's motivation and thus improving their performance. There is need for the health service managers at the county to strengthen health workers’ training by ensuring trainings are more regular and involve them in discussing their career development prospects. Future studies should include health workers from private institutions in order to incorporate their views in order to adequately inform future interventions and other sub counties in the entire county of Narok should also be included.

## Conclusion

Although results pointed out an average level of motivation among health workers in Narok County, the findings also indicated that variations in motivation levels depended on different health care system environments which indicate level of implementation of training intervention. On-job training had been offered to health workers in Narok County and had an impact of motivating the workforce as well as enhancing their performance. The organization had staff training and development policy, opportunities for career development were available, appropriate training was conducted to ensure health workers carry out their duties well, in-service training provided was adequate in dealing with existing skills gap, and that health care workers participated in identifying their career development needs. Most significant is that there was an agreement between health workers and health services managers as to the influence of training on health worker's motivation leading to improved performance. Additionally, there was a relationship between training and motivated health workforce in Narok County as training significantly predicted general motivation, job satisfaction, intrinsic job satisfaction and organizational commitment. The findings of this study have implications for all the stakeholders involved in the management of healthcare at the county and sub county levels. The county health management systems as well as health partners need to discuss the issues and adopt recommendations raised by this study. If this is done, it is hoped that key issues that are brought forward by this study will be used as a basis for improving current training intervention in order to have a motivated health workforce that can help drive the health agenda forward.

### What is known about this topic

- Provision of continuing education and development is universally known to be inadequate especially in Low and Middle Income Countries (LMICs) where the quality of basic training of health professionals varies widely leading to low skills levels among health workers.
- Good-quality training/continuing professional development on the other hand, is known to be a positive incentive which motivates health workers leading to improved performance.
- Training is known to be essential in equipping a workforce with the right skills, knowledge and capability to maximize performance.

### What this study adds

- There was an average level of motivation among health workers in Narok County.
- On-job training had been offered to health workers in Narok County and had an impact of motivating the workforce as well as enhancing their performance.
- There was a relationship between training and motivated health workforce in Narok County as training significantly predicted general motivation, job satisfaction, intrinsic job satisfaction and organizational commitment.
